# Isolation, Structural Characterization, and Hypoglycemic Activities In Vitro of Polysaccharides from *Pleurotus eryngii*

**DOI:** 10.3390/molecules27207140

**Published:** 2022-10-21

**Authors:** Pin Gong, Hui Long, Yuxi Guo, Siyuan Wang, Fuxin Chen, Xuefeng Chen

**Affiliations:** 1 School of Food and Biological Engineering, Shaanxi University of Science and Technology, Xi’an 710021, China; 2 School of Chemistry and Chemical Engineering, Xi’an University of Science and Technology, Xi’an 710054, China

**Keywords:** *Pleurotus eryngii*, polysaccharides, chemical characterization, hypoglycemic activities, PI3K-AKT signaling pathway

## Abstract

*Pleurotus eryngii* (PE) is an edible mushroom with high nutritional value. *Pleurotus eryngii* polysaccharides (PEPs) are one of the main active ingredients and manifest a great variety of biological activities. This study mainly focused on the chemical characterization and biological activities of PEPs, which were separated into two fractions (named WPS and P-1). WPS is mainly dominated by *β*-glycosidic bonds and contains *α*-glycosidic bonds, and P-1 only contains *α*-glycosidic bonds. The molecular weights of WPS and P-1 were 4.5 × 10^5^ Da and 2.2 × 10^4^ Da. The result of GC indicated that two the fractions were composed of rhamnose, arabinose, xylose, mannose, glucose, and galactose, with a ratio of 0.35:0.24:0.45:0.24:28.78:1.10 for WPS and 0.95:0.64:0.66:1.84:60.69:0.67 for P-1. The advanced structure studies indicated that the two fractions had no triple-helical structure, where WPS had a dense structure and P-1 had a loose structure. In addition, the antioxidant activity of WPS surpassed P-1, and the two fractions also exhibited a high hypoglycemic activity via inhibiting *α*-glycosidase activities and promoting the expression of PI3K-AKT signaling pathway based on in vitro assay and cell experiments.

## 1. Introduction

Diabetes is the outcome of abnormal insulin release or action in our body and is characterized by increased blood glucose levels. According to the International Diabetes Federation’s (IDF) 2019 estimation, 463.0 million adults are suffering from type 2 diabetes mellitus (T2DM), which will be increased to around 700.2 million by the year 2045 [1,2]. Hence, effective clinical therapy against diabetes has become an urgent demand. The consumption of functional foods, such as mushrooms, is a possible strategy to relieve T2DM.

Mushrooms are rich in nutrients, such as protein, polysaccharides, vitamins, minerals, and various functional factors, which play an important role in protecting human cells against diseases such as diabetes, inflammation, antiviral, cancer, and heart disease [3,4,5,6,7,8,9,10]. *Pleurotus eryngii,* a kind of *Pleurotus ostreatus*, is an edible mushroom widely cultivated in the world with high nutritional value. It contains various active ingredients such as polysaccharides, proteins, minerals, trace elements, vitamins, terpenoids, peptides, and other substances. Polysaccharides from *P. eryngii* (PEPs) are mainly manifested in biological activities, with effects including antitumor, anti-oxidation, anti-hyperlipidemia, bacteriostasis, and improvement in body immunity, and are becoming more and more popular in Europe and Asia [11,12,13,14]. Chen et al. found that PEPs can play a role in lowering blood sugar in mice [3]. Xu et al. suggested that PEPs had potential hepatoprotective and antioxidant effects on hyperlipidemia mice due to high-fat and high-cholesterol emulsion [15]. Despite some studies that have proved that PEPs have a hypoglycemic effect, the structure–activities relationship is still unclear, and the underlying mechanism of hypoglycemic effects of the purified PEPs should remain to be fully identified.

Hence, we purified crude PEP into two purified fractions and followed by chemical characterization analysis as well as in vitro and in situ hypoglycemic activities. Furthermore, the possible hypoglycemic mechanism of polysaccharides was also investigated. The purpose of this study was to expand the production and application of PEPs. Our data also provide a theoretical basis for exploring the structure–activity relationship of polysaccharides.

## 2. Materials and Methods

### 2.1. Materials and Regents

PE was purchased from Xi’an Chengxin Edible Fungus Planting Co., Ltd., Xi’an, China. DEAE cellulose, common dialysis bag (8–14 KDa), 2,2-diphenyl-1-picrylhydrazyl (DPPH, purity ≥ 98%), 4-nitrophenyl-d-pyridyl Glucosinolate (pNPG, purity ≥ 98%), and *α*-glucosidase(enzymatic activity ≥ 50 units/mg protein) were purchased from Shanghai Yuanye Biotechnology Co., Ltd., Shanghai, China. Glucose, xylose, arabinose, mannose, rhamnose, galactose, glucuronic acid, and standard were purchased from Hefei Bomei Biological Technology Co., Ltd., Hefei, China. Other chemicals used in the experiment were of analytical grade.

### 2.2. Extraction and Isolation of P. eryngii Polysaccharides

The PEPs were extracted by using hot water and precipitated with 95% alcohol. Briefly, freshly smashed fruiting bodies were dried in oven under 50 °C until the weight of fruiting bodies was unchangeable. Then, breaking up the dried fruit bodies into a uniform powder is necessary in order to increase the yield of crude polysaccharides. The powder was extracted with hot water under 40 °C (1:40, *w*/*v*) for 3 h before collected water extract was precipitated with 95% alcohol (1:4, *v*/*v*) for 24 h in refrigerator. The precipitate was separated and dissolved in 20 mL distilled water, and a Sevage method reagent (N-butanol: chloroform = 1:4, *v*/*v*) was added three times to remove the proteins. Finally, the sample was freeze-dried after dialyzing (8–14 KDa) in dissolved water for 2 days, which was named *P. eryngii* polysaccharides (PEPs). The crude polysaccharide yield of *P. eryngii* was calculated by using the following equation:

PEP yield (%) = (W2/W1) × 100%

where W2 is the weight of the crude polysaccharide and W1 is the weight of the dried PE powder.

The crude PEP was dissolved in distilled water to 10 mg/mL and purified by DEAE cellulose anion-exchange column chromatography. Subsequently, the column was eluted with NaOH, HCl, and NaOH solution in order to activate it and eluted with distilled water until pH = 7.0, followed by a stepwise elution of 0–0.5 M NaCl solubilized in deionized water at a flow rate of 5 mL/min. A total of 10 mL was collected per tube.

Two fractions (10 mL/tube) were collected according to total carbohydrate contents quantified by phenol-sulfuric acid method using an automatic fraction collector to trace the elution curve. The eluates in different concentrations were combined, concentrated in sequence, dialyzed, and freeze-dried to obtain two purified polysaccharides: distilled-water-eluting polysaccharide (WPS) and 0.1 mol/L NaCl-eluted polysaccharide (P-1). After dialysis, concentrated and lyophilized fractions were further purified with a Sephadex G-100 column (2.6 cm × 60 cm) eluted with distilled water at a flow rate of 0.3 mL/min to obtain two purified polysaccharides: distilled-water-eluting polysaccharide (WPS) and 0.1 mol/L NaCl-eluted polysaccharide (P-1).

### 2.3. Physicochemical Properties of PEP

The following indexes were determined using a UV-visible spectrophotometer L9 (Shanghai Precision Scientific Instrument Co., Ltd., Shanghai, China). Determination of total sugar content was conducted using anthrone sulfate method with D-glucose as a standard [16]. The protein content was determined by Coomassie Brilliant Blue G-250 method using bovine serum albumin as a standard. The content of uronic acid was measured by oxazole–sulfuric acid method using glucuronic acid as a standard [17].

#### 2.3.1. Total Sugar Content

Total sugar concentration in dried PS samples was determined by the colorimetric phenol–sulfuric acid method. A total of 5 mg of dried PS was dissolved in 5 mL of water, and then 400 μL of dissolved sample and 400 μL of 5% phenol solutions (*w*/*v*) were mixed with 2 mL of 95% H_2_SO_4_, strongly vortexed, and left at room temperature for 20 min. The absorbance was measured at 490 nm, and all measurements were completed in duplicate. D-(+)-Glucose was used as a standard and a calibration curve.

#### 2.3.2. Protein Content

Using the Bradford method, the protein content was determined: 0.5 mL of a 5 mg/mL sample was diluted with water to 1 mL, then 5 mL of Coomassie brilliant blue G-250 solution was added, agitated vigorously, and left for 10 min. The absorbance was measured at 595 nm. BSA was utilized as the standard ingredient to generate a standard curve, and the protein content was determined using the standard curve.

#### 2.3.3. Uronic Acid Content

We took 0.5 mL of a sample solution with a concentration of 2 mg/mL, diluted it with water to 1 mL, and performed the preceding steps at a low temperature. Next, we added 6 mL of concentrated sulfuric acid and gently shook it. We placed the mixture in an 85 °C water bath for 20 min. Each tube was filled with 0.2 mL of a 1 mg/mL carbazole solution dissolved in 95% ethanol, shaken evenly, and left at room temperature for two hours. The absorbance value was measured at 530 nm, and the standard curve containing galacturonic acid was utilized to create the standard curve. The concentration of uronic acid was determined using the standard curve.

### 2.4. Chemical Characterization of WPS and P-1

#### 2.4.1. Ultraviolet Full-Wavelength Scan of WPS and P-1

Purified polysaccharide fractions WPS and P-1 were dissolved in distilled water to the concentration of 10 mg/mL under magnetic stirring until completely solubilized. UV spectrum was recorded on a UV-visible spectrophotometer L9 (Shanghai Precision Scientific Instrument Co., Ltd., Shanghai, China). in the range of 200–400 nm every 10 nm.

#### 2.4.2. FT-IR Analysis of WPS and P-1

The purified fractions were ground with KBr powder and pressed into pellets for FT-IR measurement in the frequency range of 400–4000 nm^−1^ using an FT-IR spectrometer (PerkinElmer, Waltham, MA, USA) [18].

#### 2.4.3. 1D-NMR Analysis

A 50 mg/mL solution of polysaccharide was prepared with D_2_O as solvent. ^1^H NMR and ^13^C NMR spectrum was recorded with AV III-600 NMR spectroscopy (Bruker, Hamburg, Germany).

#### 2.4.4. Analysis of Monosaccharide Composition with GC

The monosaccharide composition analysis of WPS and P-1 was achieved using sugar nitrile acetate derivatization in GC analysis. Briefly, 20 mg of polysaccharides was hydrolyzed by 2 mL of 4 M Trifluoroacetic acid (TFA) at 110 °C for 2 h in order to release constitutive monosaccharides. Methanol was added to the system, followed by evaporation to dryness in order to remove TFA and the obtained substance became a hydrolysate. A total of 20 mg of hydroxylamine hydrochloride, 2 mg of inositol (as an internal reference), and 1 mL of pyridine were reacted with the hydrolyzate at 90 °C for 30 min. Then, 1.0 mL of acetic anhydride was added to the mixture and reacted again at 90 °C for 30 min after the sample was treated by 90 °C water bath oscillation, which was used to process the acetylation. All standard sugars (glucose, xylose, arabinose, mannose, rhamnose, galactose, and glucuronic acid) were converted to their acetylated derivatives according to the methods described above. The acetylated derivatives of WPS and P-1 were analyzed by GC.

Agilent 7890B-5977 (Agilent Technologies Inc., Santa Clara, CA, USA) was used for detection. The chromatographic conditions were: DB-5 capillary column (30 m × 0.32 nm, 0.25 μm); the inlet temperature was 250 °C; the injection volume was 1 μL; the split ratio was 10:1; the carrier gas was ultra-pure nitrogen; and the temperature of the detector was 270 °C. Column heating procedure: initial temperature 150 °C, held for 0.5 min; temperature increased to 190 °C at a rate of 7 °C/min and held for 2 min. The temperature was then raised to 250 °C at a rate of 15 °C/min and maintained for 8 min.

#### 2.4.5. Determination of Molecular Weight

The molecular weight of WPS and P-1 was determined by HPGPC according to the previous study [19], which was performed on a Shimadzu LC-20AT HPLC system (Shimadzu Co., Kyoto, Japan) with column type ShodexOHpak. Sample was eluted with distilled water (with 0.02% NaN_3_) at the flow rate of 1 mL/min with 500 μL of injection volume. T-series dextran standards were used for constructing the calibration curve.

Y = −5.3425 × e^−5^X^3^ + 1.2596 × e^−2^X^2^ − 1.0358X + 36.8901 (R^2^ = 0.9998)

where Y is the logarithm of saccharides, Mw; and X is the saccharide sample’s peak elution time.

#### 2.4.6. Morphological Analysis

The microstructure was observed using a field emission scanning electron microscope Nano G2 (Compound Scientific Instruments Co., Ltd., Shanghai, China). An appropriate number of purified polysaccharides WPS and P-1 was pasted on a metal stage, followed by coated with gold powder for 30 s. Every sample was recorded under a magnification of 400, 3000, and 12,000.

#### 2.4.7. Congo Red Experiment

The Congo red solution was added to sample at concentration of 1 mg/mL, followed by adding NaOH solution at 0.1 mol/L, 0.2 mol/L, 0.3 mol/L, 0.4 mol/L, and 0.5 mol/L. Then, the reaction system was scanned in the range of 400–600 nm band using an ultraviolet–visible spectrophotometer L9 (Shanghai Precision Scientific Instrument Co., Ltd., Shanghai, China) after 30 min. The distilled water without adding WPS and P-1 served as the control.

### 2.5. Determination of Antioxidant

#### 2.5.1. DPPH Free-Radical Scavenging Activity

A total of 150 uL DPPH (0.2 mM in absolute ethanol) with 150 μL of different concentrations (0.05, 0.1, 0.2, 0.4, 0.8, and 1 mg/mL) and sample solution was mixed in 96-well microtiter plates, followed by shaking the mixture and incubating for 30 min at room temperature in the dark. Using the same concentration, VC served as a positive reference. The absorbance of the solution was measured at 517 nm. The scavenging activity was calculated by the following equation:

DPPH free radical scavenging rate (%) = [1 − (A1 − A2)/A0] × 100%

where A0 is the absorbance of the control (using distilled water instead of the sample solution), A1 is the absorbance of the sample, and A2 is the absorbance of the sample in which the DPPH free-radical solution is replaced with absolute ethanol.

#### 2.5.2. Hydroxyl Radical-Scavenging Activity

A total of 100 uL of FeSO_4_ solution (6 mM) and 100 μL of salicylic acid–ethanol solution (6 mM) was added to sample solution at different concentrations (0.05, 0.1, 0.2, 0.4, 0.8, and 1 mg/mL). Subsequently, the reaction system was triggered by adding 25 μL H_2_O_2_, and the mixture was incubated for 30 min at 37 °C. The absorbance of the mixture was measured at 510 nm after cooling down to room temperature, with positive reference to the same concentration of VC. The scavenging activity was calculated by the following equation:

Hydroxyl free scavenging rate (%) = [1 − (A1 − A2)/A0] × 100%

where A0 is the absorbance of the control (using distilled water instead of the sample solution), A1 is the absorbance of the sample, and A2 is the absorbance of the sample using distilled water instead of H_2_O_2_.

#### 2.5.3. Superoxide Anion-Scavenging Activity

A total of 3 mL of Tris-HCl solution (pH = 8.2) was added to 1 mL of sample solution at different concentrations (0.05, 0.1, 0.2, 0.4, 0.8, and 1 mg/mL) before 30 °C water baths for 20 min. Subsequently, 3 mL pyrogallic acid solution (5 mM) was mixed into the reaction system, followed by shaking for 3 min and the addition of 1 mL of concentrated hydrochloric acid for termination. Using the same concentration, VC served as a positive reference. The absorbance of the solution was measured at 320 nm.

peroxide anion scavenging rate (%) = [1 − (A1 − A2)/A0] × 100%

where A0 is the absorbance of the control (using distilled water instead of the sample solution), A1 is the absorbance of the sample, and A2 is the absorbance of the sample using distilled water instead of pyrogallic acid.

### 2.6. Determination of Hypoglycemic Activity

#### 2.6.1. Determination of In Vitro α-Glucosidase Inhibitory Activity

A total of 40 μL of sample containing different concentrations of WPS and P-1 (0.5, 1, 2, 4, and 8 mg/mL) was mixed with 40 μL of pH 6.8 sodium phosphate buffer (0.1 mol/L) and 20 μL of *α*-glucosidase solution (0.8 U), and the mixture was incubated for 15 min at 37 °C, followed by the addition of 20 μL of PNPG solution (10 mM) in order to catalyze the reaction. Finally, the mixture was incubated for 20 min at 37 °C after shaking well. Then, the absorbance was measured at 405 nm. Using acarbose as a reference, the inhibitory activity was calculated by the following equation:

*α*-Glucosidase inhibition rate (%) = [1 − (D_1_ − D_2_)/D_0_] × 100%

where D0 is the absorbance of the control (using distilled water instead of the sample solution), D1 is the absorbance of the sample, and D2 is the absorbance of the addition of sodium–phosphate buffer without *α*-Glucosidase.

#### 2.6.2. Cell Culture and IR-HepG2 Cell Model Construction

HepG2 cells were obtained from the Xi’an Medical College (Xi’an, China). Cells were cultured in DEME medium supplemented with 10% heat-inactivated FBS and 1% mixture of penicillin and streptomycin in a humidified incubator at 37 °C with 5% CO_2_. Cells in the logarithmic growth phase were digested and cultured in the 96-well microplates (1 × 10^5^ cells/mL). A total of 200 μL of insulin mother liquor (insulin powder dissolved in diluted hydrochloric acid diluted with distilled water until pH 2–3) in different concentrations (10^−5^, 10^−6^, 10^−7^, 10^−8^, and 10^−9^ mmol/L) was added to wells containing cells after incubating for 24 h. Then, 24 h, 48 h, and 72 h incubation for each concentration was used to create insulin-resistance model; the supernatant was collected by centrifuging for 10 min and then adding it to another new 96-well microplate. Subsequently, 300 μL of glucose oxidase assay kit assay solution and 3 μL of the centrifuged supernatant were added to each group in order, and the absorbance was measured by using Varioskan Flash (Thermo Fisher, Waltham, MA, USA) at 505 nm after 15 min of incubation. In addition, the calibration group (using calibration solution instead of supernatant) and ultrapure water group (using ultrapure water instead of supernatant) were added for contrast.

The glucose remaining was calculated by the following equation:

Glucose remaining = Sample-Blank/Calibration-Blank × 5.55

#### 2.6.3. Effect of PEP on IR-HepG2 Cells

The detection of glucose intake was performed following the previous methods with sight modifications [20]. The cells were planted in the 96-well plate at a concentration of 1 × 10^5^ cells/mL. Control group was maintained in DMEM medium; metformin was chosen as the positive control. In the treatment group, WPS and P-1 at a level of low, medium, and high dose were added to the well, which contained culture medium with suitable concentration of insulin, and incubated. Additionally, the remaining glucose was measured.

#### 2.6.4. Western Blot

Protein expression of insulin-related PI3K-AKT signal pathway was determined using Western blot, as in a previous study, with minor modification [21]. Briefly, total protein was extracted from HepG2 cells by RIPA lysis using a BCA protein assay kit (Beyotime, Shanghai, China), followed by preparation of separation gel, concentrated gel, and protein separation. Protein was subjected to SDS-PAGE, transferred to PVDF membranes (Millipore, Billerica, MA, USA), and washed with buffer for 4 floods. Subsequently, the PVDF membranes were blocked with blocking buffer for 1 h at 37 °C. The PVDF membranes were incubated at 4 °C overnight with the primary antibody solutions, including GAPDH, PI3K, and AKT (Sangon Biotech Co., Ltd., Shanghai, China), followed by incubating with anti-rabbit antibodies conjugated to alkaline phosphatase for 1 h at 37 °C. The membranes were washed by TBS-Tween 20 four times after each incubation. Proteins were visualized using ChemiDox gel imaging system to take pictures.

### 2.7. Statistical Analysis

The data of samples were calculated using Origin software 8.0. All data were presented as means ± standard deviation (means ± SD) and calculated using one-way ANOVA of SPSS 17.0 followed by Tukey’s multiple-range test. The statistical significance was defined as *p* < 0.05 or *p* < 0.01.

## 3. Results

### 3.1. Separation and Purification of PEP

**Figure 1:**
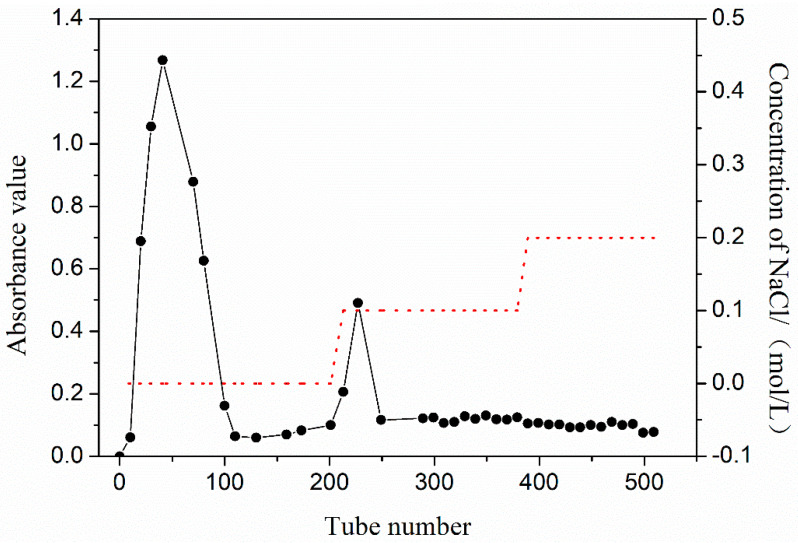
Elution curve of crude PEP using different concentrations of NaCl.

The yield of crude PEP extracted from the mushroom powder was approximately 5.9%. The crude PEP was then firstly purified through a cellulose DEAE anion-exchange column. The elution curve obtained by eluting with different concentrations of NaCl is shown in Figure 1. As a result, two fractions were collected with a stepwise concentration (0, 0.1 mol/L) of NaCl solution, and were named WPS and P-1, respectively.

### 3.2. Determination of Certain Chemical Components in PEP

The total sugar content of WPS was 87.87%, and that of P-1 was 73.88%. There were a few proteins among WPS and P-1 (0.02% and 1.27%). The concentration of uronic acid of WPS and P-1 was 2.11% and 6.46%, respectively.

### 3.3. Ultraviolet Full-Wavelength Scanning

The ultraviolet full-wavelength scanning spectra of WPS and P-1 are shown in Figure S1. The absorption peaks of nucleic acids and proteins at 260 nm and 280 nm of WPS and P-1 were extremely weak, indicating that the content of nucleic acids and proteins in the sample after separation and purification was significantly decreased.

### 3.4. FT-IR Analysis

Through FT-IR studies, functional groups of WPS and P-1 were monitored within the range of 500–4000 cm^−1^ (Figure S2). As a result, the broad absorption peaks at 3455 cm^−1^ were related to the O-H hydroxyl groups, with stretching vibration in the sugar ring [22]. The peaks at around 2937 cm^−1^ and 1415 cm^−1^ suggested the presence of stretching vibration and bending vibration in the bond of C-H [23,24]. In addition, the peaks presented at around 1642 cm^−1^ were assigned to C=O stretching vibration [25]. The peak near 1078 cm^−1^ indicated that two fractions might have a pyran ring. The absorption peak at around 1037.96 cm^−1^ was caused by C-O-C stretching vibration [26]. The appearance of the absorption band near 918 cm^−1^ means that two fractions’ molecules contained *β*-configuration glycosidic bonds [27]. Compared with the finding reported by Ma et al. (2014), there were three different PEPs, named PEPE-1, PEPE-2, and PEPE-3, whose peaks signals at around 2360 and 2340 cm^−1^ were the characteristic absorptions of aliphatic C-H bonds and C≡N groups, and the signal 1250 cm^−1^ was assigned to the stretching vibration of C=O groups [28]; however, WPS and P-1 did not show the similar signals, indicating that the differences in functional groups were probably due to the different sources of PE.

### 3.5. NMR Analysis of WPS and P-1

Figure S6 illustrates the δ5.4 ppm ^1^H-NMR spectra of WPS and P-1. There was no discernible signal, indicating that both polysaccharides were pyranose. Typically, they fall between δ4.3 ppm and δ5.6 ppm. Polysaccharides characterize glycosidic bond-type regions. WPS contained two heterocephalic hydrogen signals between δ4.3 ppm and δ4.9 ppm, indicating the presence of β-glycosidic bonds, and the signal at δ4.7 ppm was stronger than the signal at δ5.02 ppm, indicating that WPS was a polysaccharide with β-configuration glycosidic bonds. P-1 had a significant proton signal only at δ4.7 ppm, indicating that its glycosidic bonds were only of the β-configuration.

In the analysis of ^13^C-NMR of WPS, the signal of C3 and C5 between δ82 ppm and δ88 ppm is the furan ring, whereas the signal below δ80 ppm is the pyran ring. The absence of a heteroheaded carbon signal at δ82 pm~δ88 pm further demonstrates that the sugar residues in polysaccharide WPS are of the pyryl type, which is consistent with the preceding ^1^H-NMR spectrum. C-1 is present between δ95 ppm and δ110 ppm, whereas the signal distribution of C-1-C-2 is between δ60.36 ppm and δ72.37 ppm. At δ > 170 ppm, there was no carbon signal, indicating that polysaccharide WPS did not include uronic acid and was a neutral polysaccharide, consistent with the prior infrared spectrum investigation.

### 3.6. Analysis of Monosaccharide Composition by GC

The gas chromatograms of WPS and P-1 are shown in Figure S3. According to the retention time and response intensity, the molar ratio of each monosaccharide was calculated by comparing it with various standard samples. The data showed that WPS was composed of rhamnose, arabinose, xylose, mannose, glucose, and galactose, with a ratio of 0.35:0.24:0.45:0.24:28.78:1.10, and the monosaccharide composition of P-1 was the same as WPS but with different ratio of 0.95:0.64:0.66:1.84:60.69:0.67.

### 3.7. Molecular Weight (Mw)

Taking different *Mw* dextran as the standard sample, according to their mass distribution and the spectrum shown in Figure S4, the *Mw* of WPS and P-1 were 4.5 × 10^5^ Da and 2.2 × 10^4^ Da.

### 3.8. Morphological Analysis

**Figure 2:**
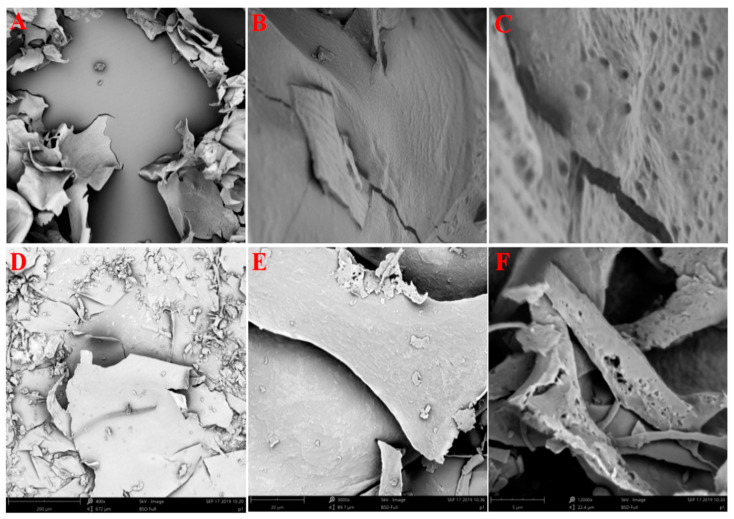
SEM images of PEPs (WPS: (**A**–**C**)) and (P-1: (**D**–**F**)). ((**A**,**D**): ×400; (**B**,**E**): ×3000; (**C**,**F**): ×12,000).

The spatial structure of polysaccharides can affect the structure–activity relationship. Scanning electron microscopy (SEM) could be set as a characteristic to qualitatively identify the surface morphology of polysaccharides. SEM of two polysaccharides, WPS and P-1, is shown in Figure 2. The dense and flat micro-surface of WPS, which can be observed as a relatively large and irregular sheet structure after magnification, suggested that the sugar chains were tightly clustered due to their large molecular weight. The surface of P-1 was relatively loose, with many irregular fragments under the 400× lens. As the magnification is increased to the 12,000× lens, the loose and porous network structure can be better observed, which resulted from a lack of tight aggregation of polysaccharide chains as well as the lower molecular weight compared to WPS.

### 3.9. Congo Red Experiment

Congo red could combine with polysaccharides possessing an ordered three-dimensional structure into a complex in the solution, and the maximum absorption wavelength in the spectral scan would shift when adding NaOH with different concentrations to the solution [29]. The result of the Congo red experiment is shown in Figure S5. When the NaOH concentration was beyond 0.2 mol/L, the maximum absorption wavelength of the polysaccharide and Congo red complex did not decrease significantly compared with the maximum absorption wavelength of Congo red dye, showing that there was no triple helix structure in polysaccharides due to no unwinding appearing as the NaOH concentration increased.

### 3.10. Antioxidant Activity of WPS and P-1

**Figure 3:**
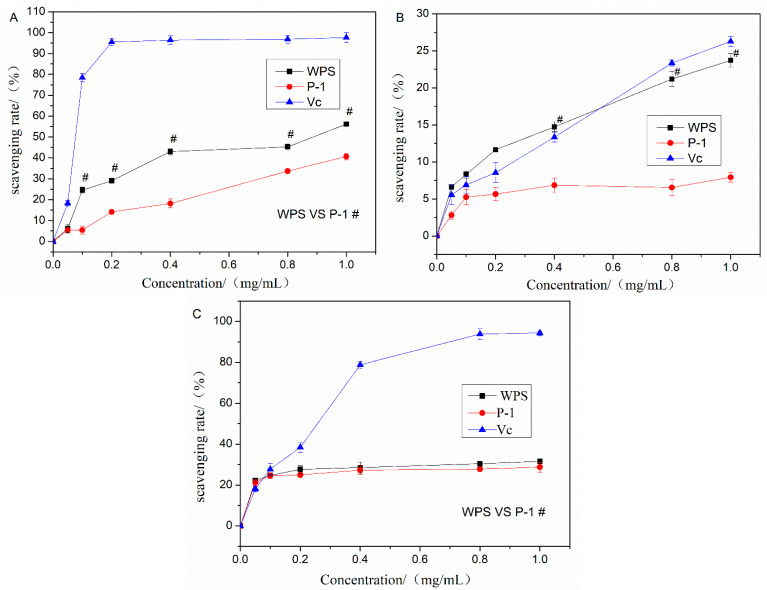
In vitro antioxidative effect of PEPs (WPS and P-1). (**A**) DPPH scavenging test. (**B**) Hydroxyl radical scavenging test. (**C**) Superoxide anion scavenging test. The data are presented as means ± SD (*n* = 3). ^#^ *p* < 0.05, compared with P-1.

Free radical production and elimination in the human body is a dynamic balance. Once the balance has been disrupted, excessive free radicals in the body can induce damage to cells and tissues, leading to various diseases and accelerated body aging. This study evaluated the antioxidant activities of WPS and P-1 using three different in vitro methods. The results are shown in Figure 3A. WPS and P-1 had a certain scavenging effect on DPPH with a dose–effect relationship in the range of certain concentrations. WPS had better scavenging ability than P-1 in the range of certain concentrations. When the concentration reached 1 mg/mL, the scavenging ratio of WPS, P-1, and VC were 56.21%, 40.68%, and 97.63%, respectively. This showed that WPS and P-1 had a certain scavenging effect on DPPH but were lower than VC at the same concentration.

Figure 3B showed that the hydroxyl radical-scavenging ability of WPS and VC had concentration dependence excluding P-1. When the concentration reached 0.8 mg/mL, the scavenging rate exceeded VC. The scavenging effect of WPS was significantly higher than that of P-1, probably due to the existence of *α*-glycoside bond in WPS, which is consistent with the result reported by Chen et al. [30].

In this study, the superoxide anion-scavenging ability of WPS is shown in Figure 3C. Two fractions, WPS and P-1, had a certain scavenging effect on superoxide anion free radicals, but the two polysaccharides did not show a good dose–effect relationship within this concentration range. When the two fractions reached a concentration of 0.1 mg/mL, the scavenging rate for superoxide anion free radicals tended to be stable. However, the scavenging effect was much lower than VC at the same concentration.

### 3.11. Effects of WPS and P-1 on α-Glucosidase Activities

**Figure 4:**
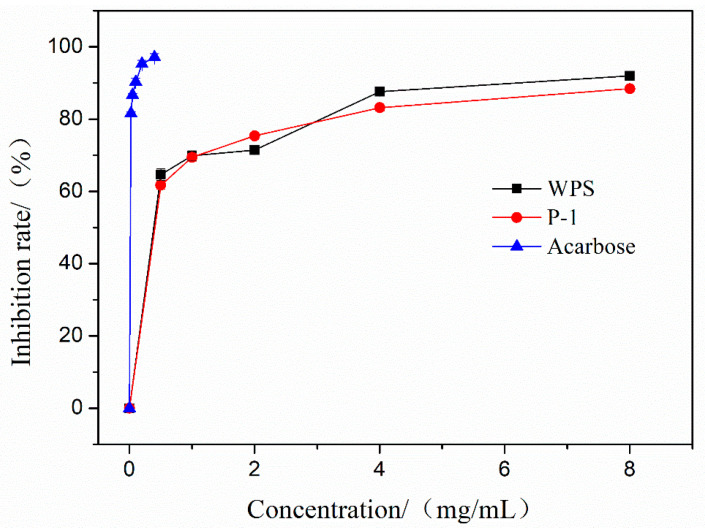
α-glucosidase inhibition test of PEPs (WPS and P-1).

The inhibitory effects of WPS and P-1 on the activities of *α*-glucosidase are shown in Figure 4. WPS and P-1 showed dose-dependent inhibitory effects on *α*-glucosidase activity. Especially at the high concentration of 8 mg/mL, the highest inhibitory rates of WPS and P-1 were 91.95% and 88.45%, and IC50 were 0.4211 mg/mL and 0.2971 mg/mL, respectively. At each concentration point, the inhibitory activities of WPA and P-1 were similar. As the positive control, acarbose showed a higher inhibition rate under a lower concentration.

### 3.12. Effect of WPS and P-1 on IR-HepG2 Cells

The two polysaccharide fractions had a certain promotional effect on cell growth within a concentration of 20~640 μg/mL in a 48 h time period without obvious toxic effect on the HepG2 cells. According to their different effect on cell viability in this experiment, the concentrations of 40 μg/mL, 80 μg/mL, and 160 μg/mL for WPS were chosen for 48 h as well as 160 μg/mL, 320 μg/mL, and 640 μg/mL for P-1 for 48 h.

Insulin resistance was formed due to various reasons, most likely probably because the release of insulin cannot normally promote the uptake and utilization of glucose for cells, so the secretion of excessive insulin occurs to maintain normal blood sugar levels, finally resulting in the occurrence of type 2 diabetes and other metabolic syndromes [31]. In this study, the glucose consumption of HepG2 cells stimulated by insulin at different concentrations and different time periods was tested to optimize the constructing conditions of the IR model. The glucose consumption of HepG2 cells was reduced after stimulating with 1 × 10^−7^ mol/L insulin for 48 h, showing a significant difference compared with the control group (*p* < 0.01). Therefore, the final modeling conditions were selected as treatment with 1 × 10^−7^ mol/L insulin concentration for 48 h.

**Figure 5:**
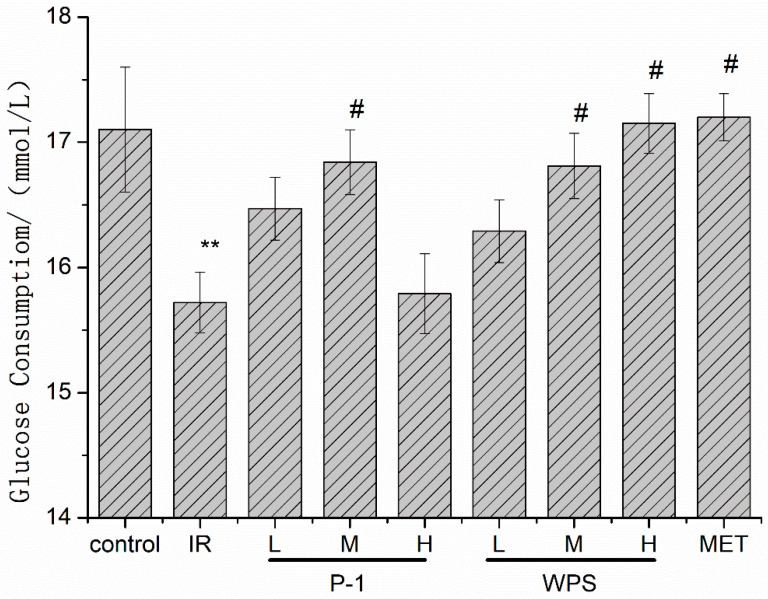
Effect of PEPs (WPS and P-1) on IR-HepG2 cell glucose consumption. The data are presented as means ± SD (*n* = 3). # *p* < 0.05, compared with control group; ** *p* < 0.01, compared with IR group.

Figure 5 indicates that WPS has a certain improvement effect on cellular insulin resistance in concentrations of 40 μg/mL and 160 μg/mL, which can be reflected in promoting the absorption of glucose, close to the Metformin group (MET), especially at a high dose of 160 μg/mL compared to the model IR group (*p* < 0.05). Concentrations of 160 μg/mL and 320 μg/mL of P-1 can also promote the consumption of glucose in the medium by cells, indicating that it had a certain improvement effect on cellular insulin resistance, especially at a medium dose, and represented a significant difference compared to model (*p* < 0.05). The effect of WPS shows a dose–effect relationship, but P-1 did not.

### 3.13. Effect of WPS and P-1 on the Protein Expression of PI3K-AKT Signaling Pathway in IR-HepG2 Cells

**Figure 6:**
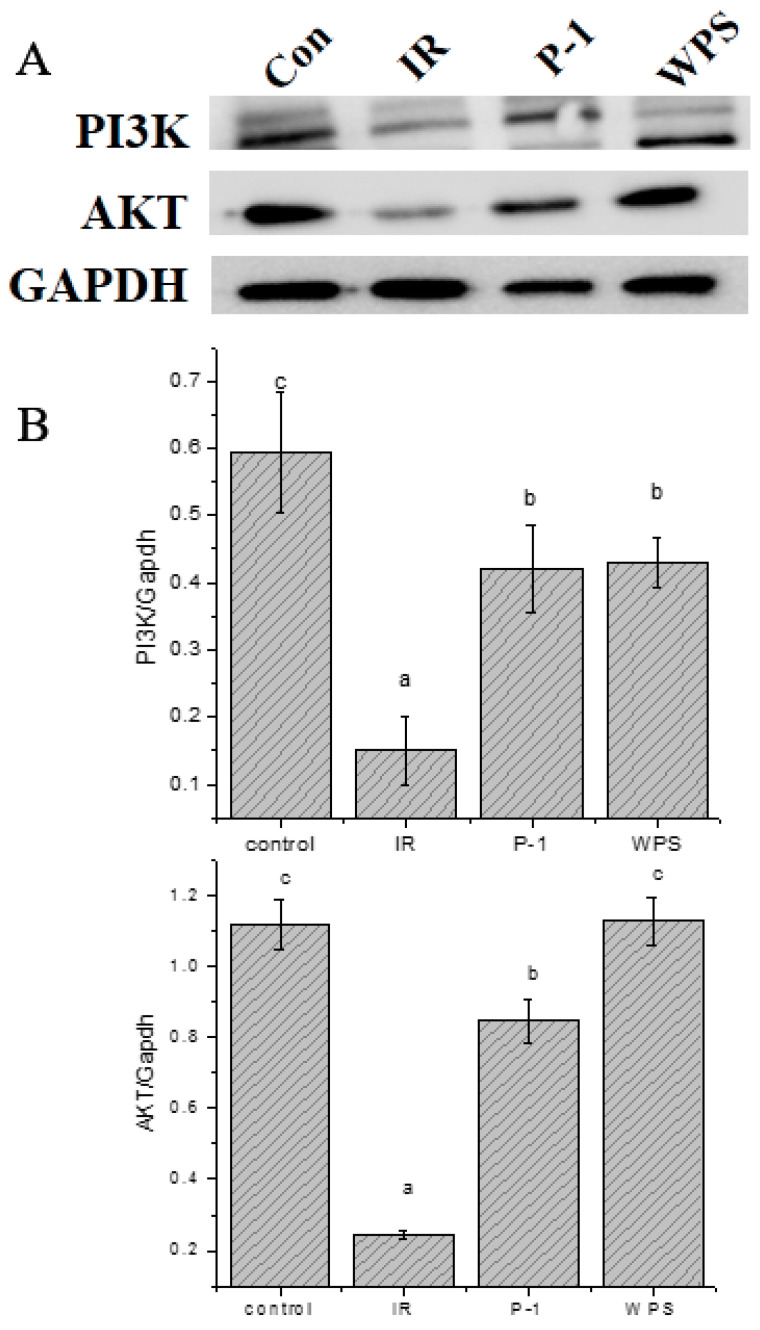
(**A**) Effects of PEPs (WPS and P-1) on protein expression of PI3K and Akt in IR-HepG2 cells. (**B**) Quantitative analysis of protein expression in different groups. Different letters indicate significant differences (*p* < 0.05).

It is widely believed that the PI3K/Akt signaling pathway plays a key role in insulin signal transduction [32,33]. In a previous study, cells developed significant insulin resistance after 48 h of high-concentration insulin stimulation. Therefore, the expression of major proteins in the glucose transport pathway was studied to determine whether polysaccharide treatment affected the PI3K/Akt signaling pathway in insulin-resistant cells. As shown in Figure 6, the insulin-resistant cells treated with WPS and P-1 significantly increased the protein-expression levels of Akt and PI3K compared with the model IR group (*p* < 0.05), which confirmed that *P. eryngii* polysaccharides WPS and P-1 can effectively activate the PI3K-Akt signaling pathway to alleviate insulin resistance.

## 4. Discussion

Mushrooms have already been valued as edible or medicinal resources worldwide [9,34]. As an important functional compound in mushrooms, polysaccharides have been extensively investigated in past years [35]. However, the structure–activity relationship, as well as the action mechanism behind the biological activity of polysaccharides, are still undetermined.

In our study, we extracted and purified two novel heteropolysaccharides, WPS and P-1, from PE. Based on the published literature, it has been observed that the difference in the chemical characteristics of PEPs may be due to the different procedures of preparation, extraction, and purification, as well as the source (mycelia or fruiting bodies) [36,37,38]. Using hot water extraction, branched (1→3)(1→6)-*β*-glucan [38], mannogalactan [39], and heteropolysaccharides [11] were extracted and purified from PE fruiting bodies. Zhang Bingru et al. [40] discussed the extraction/purification, structural characterization, and pharmacological properties of polysaccharides derived from *Pleurotus eryngii*. The polysaccharide-extraction procedure mentioned in their article is almost identical to the one utilized in our investigation. First, hot water extraction, then ethanol precipitation, and finally, the Sevage technique are used to remove proteins. Similar to our research, they felt *Pleuinus eryngii* polysaccharide had a hypoglycemic impact. However, the structure and pharmacological actions of polysaccharides have not been sufficiently examined, and require additional research.

Mushroom-isolated polysaccharides exhibit a variety of biological functions [41]. WPS and P-1 produced after purification in this investigation show substantial antioxidant and α-glucosidase-inhibiting properties, making them promising biological macromolecules. Although Rizkyana A.D. et al. [42] extracted polysaccharides using subcritical water, its high cost precluded large-scale promotion and hampered its development. Gunasekaran S’s team extracted the acidic polysaccharide from *Pleurotus eous* (*Berk.*) and modified it with sulfation to improve its antioxidant, antibacterial, and anticoagulant properties [43,44]. Due to the complexity of polysaccharides, the reproducibility and safety of introducing new groups are deserving of further study, but hot-water extraction is more secure and has a broader range of practical applications.

The existence of uronic acid residues in polysaccharides may affect the characteristics of polysaccharides and alter the solubility of associated polysaccharide conjugates. Therefore, it is important to assay the content of uronic acids for the quantitative and structural analysis of complex carbohydrates [45]. The contents of uronic acid in WPS and P-1 were measured, and the results indicated that the contents of uronic acid in WPS and P-1 were low, 2.11% and 6.46%, respectively, suggesting that WPS and P-1 may be neutral polysaccharides.

The WPS and P-1 polysaccharides were extracted using water and eluted with water and 0.1 mol/L NaCl, respectively. The method of extraction had an effect on the structure of polysaccharides [46]. Ren, D. et al. extracted two polysaccharides, PEP-1 and PEP-2, with distilled water at 80 °C and eluted them with distilled water and 0.05 mol/L NaCl. The monosaccharide components of both polysaccharides were mannose, glucose, and galactose, with glucose constituting approximately 49.21% of the polysaccharide, and the molecular weights were 2.54 × 10^4^ Da and 4.63 × 10^5^ Da, respectively [11]. It is evident that the molecular weights of the two polysaccharides are quite distinct. Consequently, it is crucial to investigate the molecular weight of polysaccharides [47]. β-glucan may be the active structure of polysaccharides, which has some effect on polysaccharide activity [36,48]. In this study, FT-IR analysis revealed an absorption band near 918 cm^−1^, which indicates that two fractions’ molecules contained configuration glycosidic bonds, thereby indicating that WPS and P-1 polysaccharides have beneficial physiological activities.

Data obtained from in vitro antioxidant assays demonstrated that WPS and P-1 showed different antioxidant activities in different reactive systems, probably due to their specific structures and extraction methods. Zhang et al. (2014) applied ultrasonic technology to extract polysaccharides from the fruiting bodies of PE and found that the concentration of these crude polysaccharides was positively correlated with the DPPH free-radical scavenging rate and superoxide anion-radical scavenging ability; PEPS80 had the best antioxidant activity [49]. Li et al. (2016) also demonstrated that FDPEPS from PE, after being treated with heat, might donate more electrons or act as a more efficient electron donor than other polysaccharides, probably because heat processing may cause irreversible modifications on the original structures of polysaccharides [6]. We discovered that WPS exhibited superior antioxidant activity to P-1. There were significant differences in their hydroxyl radical scavenging abilities but no obvious differences in their superoxide anion-scavenging abilities.

The inhibition of digestive enzymes such as *α*-glucosidase is considered to be a promising therapy to treat T2DM [50]. The effect of *α*-glucosidase can degrade oligosaccharides into monosaccharides, resulting in an increase in blood glucose levels. Therefore, *α*-glucosidase has been known as a therapeutic target for the modulation of postprandial hyperglycemia. In this study, the inhibitory effects of WPS and P-1 on *α*-glucosidase were measured, and WPS and P-1 showed certain inhibitory effects on *α*-glucosidase, indicating that WPS and P-1 could be a potential inhibitor for *α*-glucosidase used in functional food. The inhibition rate of WPS and P-1 can reach 80%, but compared with acarbose, the concentration of WPS and P-1 is larger, and its IC50 value is larger.

Diverse research has been focused on the antidiabetic effect of PEPs on animal models. Ren et al. demonstrated that PEPs, a heteropolysaccharide extract from PE, are capable of attenuating the development of insulin resistance and oxidative stress in HF-fed mice [51]. PEPs also increased the level of high-density lipoprotein cholesterol and liver glycogen in KKAy mice [3]. Although PEPs have been demonstrated to have a hypoglycemic effect, fewer studies have focused on their mechanism. To further elucidate the hypoglycemic effect of WPS and P-1, glucose consumption in HepG2 cells was investigated. Insulin resistance (IR) is a serious risk factor leading to the onset of T2DM, and ameliorating the IR status is probably an effective approach for the treatment of T2DM [52]. HepG2 is a good cell model to study IR pathogenesis and the mechanism of hypoglycemic drugs in vitro because of its similar response as hepatocytes when correlated with the insulin level and the duration of stimulation [53,54]. As a result, the IR-HepG2 cells model was established as a cellular model to verify the hypoglycemic effect of WPS-1 and P-1 due to its low glucose consumption, which mimics the symptoms of T2DM. Metformin has been widely used for the therapy of T2DM, the major hypoglycemic effects of which are reduced hepatic glucose and increased insulin-stimulated glucose uptake in skeletal muscle and adipocytes [55]. Therefore, metformin was chosen as a positive control. The results of the MTT assay showed that WPS and P-1 had no toxic influence on the viability of both HepG2. After being treated with different concentrations of WPS and P-1, the two polysaccharides can indeed increase the glucose consumption of IR- HepG2 cells, which can be achieved by activating the activity of certain insulin protein receptors to restore the sensitivity of cells to insulin action and increasing glucose uptake in liver, so that the effect of corresponding concentrations of insulin can reach the level of cell metabolism as well as improve the cell’s ability to increase glucose utilization. These results indicated that WPS and P-1 possibly possess a remarkable hypoglycemic effect in vitro.

The PI3K-Akt signaling pathway is the main effector of insulin and is closely related to glycogen synthesis in the liver [56], so the highly relevant PI3K-AKT insulin signaling pathway was selected for subsequent preliminary research. The PI3K-AKT signaling pathway is related to the regulation of cell growth and metabolism, and the down-regulation of related proteins on the pathway may produce a pathological response in order to activate the related proteins such as PI3K to convert phosphatidylinositol diphosphate (PIP2) to PIP3, which has a role of gathering AKT and other kinases on the cell membrane to phosphorylate other kinases on the membrane. After, AKT can regulate other glycogen synthesis kinases, etc., to promote the synthesis of glycogen on the membrane by sugar transporters, thereby regulating the transport of glucose and playing a role in reducing glucose. The results showed that the protein level of the insulin signaling pathway PI3K-AKT in the H-WPS group and M-P1 group with the best concentration group significantly increased, which indicated that PEPs could improve insulin resistance by mediating the PI3K-AKT signaling pathway.

Generally speaking, the bioactivities of most natural polysaccharides are influenced by their molecular weight and structural features, such as anomeric carbon configuration, linkage patterns, monosaccharide compositions, sequence of sugar units, degrees of polymerization, and branching characteristics [57]. Among these factors, the molecular weight of native polysaccharides can significantly affect the biological activities and utilization of polysaccharides [49,57]. In general, higher molecular weights of polysaccharides were associated with better activities because large-molecular-weight polysaccharides may have more repetitive units in the chain, and these repetitive units may be the targets for interaction with receptors [58,59]. It was reported that two heteropolysaccharides (PEP-1 and PEP-2) from PE fruiting bodies and the higher-molecular-weight PEP-2 had higher antitumor activity on human hepatoblastoma HepG-2 cells [11]. Ma and colleagues found that PEPE-3 from PE residue with larger molecular weights had better antitumor activities, suggesting that the antitumor activities of these three polysaccharides were related to their molecular weight, monosaccharide composition, and sulfate and uronic acid content [28]. Li et al. also demonstrated that a higher-molecular-weight polysaccharide (ZSP4b) with a similar composition to a JSXZ polysaccharide (ZSP2) displayed higher antioxidant activities than ZSP2 because its higher molecular weight allowed the spatial conformation of the JSXZ polysaccharides to be maintained [60]. Herein, the antioxidant effect and hypoglycemic activity of WPS, whose molecular weight is greater, were better than P-1, probably due to their greater molecular weight. Glycosidic bonds also affect the activities of polysaccharides in mushrooms; linear *β*-glycosidic bond configuration polysaccharide has better activity than *α*- glycosidic bond configuration [61]. Furthermore, it was suggested that a certain relationship might exist between its activity and monosaccharides’ composition [62]. Human cells can recognize such carbohydrates through monosaccharide receptors, thus stimulating cytokine production. The affinity binding between the polysaccharides and the receptors is the critical step for the signaling pathway’s initiation; in other words, the triggering of the polysaccharides’ function [63,64]. Cao et al. demonstrated that polysaccharide fractions (TSP-1) from *Toona sinensis* (Juss.) *M.Roem.* leaves presented higher in vivo hepatoprotective activity, probably due to the higher contents of glucose [65]. In our study, both WPS and P-1 have extremely complicated structures composed of six monosaccharides; amongst them, glucose comprises the largest proportion. In conclusion, the antioxidant and hypoglycemic activities of PEPs are affected by their molecular weight and structural features, such as monosaccharide compositions and anomeric carbon configuration.

## 5. Conclusions

Two novel fractions, WPS and P-1, were successfully separated from crude PEPs; both WPS and P-1 consist of rhamnose, arabinose, xylose, mannose, glucose, and galactose as their monosaccharide components and may be two neutral polysaccharides with β-glycosidic linkages and different molecular weights. WPS exhibited better antioxidant and hypoglycemic activities than P-1, probably because of higher molecular weight and *β*-glycosidic bond. Both of the two fractions could exert hypoglycemic activities by quenching ROS, inhibiting the *α*-glucosidase enzyme, and activating the PI3K-AKT signaling pathway. As a result, the possible hypoglycemic mechanisms of the two polysaccharides, WPS and P-1, might be based on three factors: (1) the restraint of *α*-glycosidase enzymes; (2) the increased glucose use of hepatic cells; (3) free-radical scavenging. Our study may facilitate the utilization of *P. eryngii* as the candidate drug in clinical therapy against diabetes; however, further study on the structure–effect of PEPs is still necessary.

## Data Availability

Not applicable.

## Supplementary Materials

The following supporting information can be downloaded at: https://www.mdpi.com/article/10.3390/molecules27207140/s1. Figure S1. Ultraviolet full-wavelength scanning of PEP (WPS and P-1). Figure S2. IR spectra of different PEPs (A) WPS. (B) P-1. Figure S3. GC of PEPs (A)WPS. (B) P-1. (C) GC of mixed standard monosaccharide. 1. rhamnose; 2. arabinose; 3. xylose; 4. mannose; 5. glucose; 6 galactose; 7. internal standard. Figure S4. Gel Permeation Chromatography (GPC) spectrum of PEPs (A) GPC spectrum of WPS. (B) GPC spectrum of P-1. (C) GPC spectrum of glucan. Figure S5. Congo red test of PEPs (WPS and P-1).
