# Supplementary material for: Isolation, Structural Characterization, and Hypoglycemic Activities In Vitro of Polysaccharides from Pleurotus eryngii
Source: Molecules. 2022 Oct 21;27(20):7140. doi: 10.3390/molecules27207140 (PMC9609144; doi:10.3390/molecules27207140)
Supplement: Supplementary file 1 [file molecules-27-07140-s001.zip › molecules-1959126-supplementary.pdf]

Supplementary Materials

Isolation, Structural Characterization, and Hypoglycemic Activities In Vitro of Polysaccharides from *Pleurotus eryngii*

Pin Gong [1,†], Hui Long [1,†], Yuxi Guo [1,†], Siyuan Wang [1], Fuxin Chen [2] and Xuefeng Chen [1,*]

[1] School of Food and Biological Engineering, Shaanxi University of Science and Technology, Xi'an 710021, China
[2] School of Chemistry and Chemical Engineering, Xi'an University of Science and Technology, Xi'an 710054, China

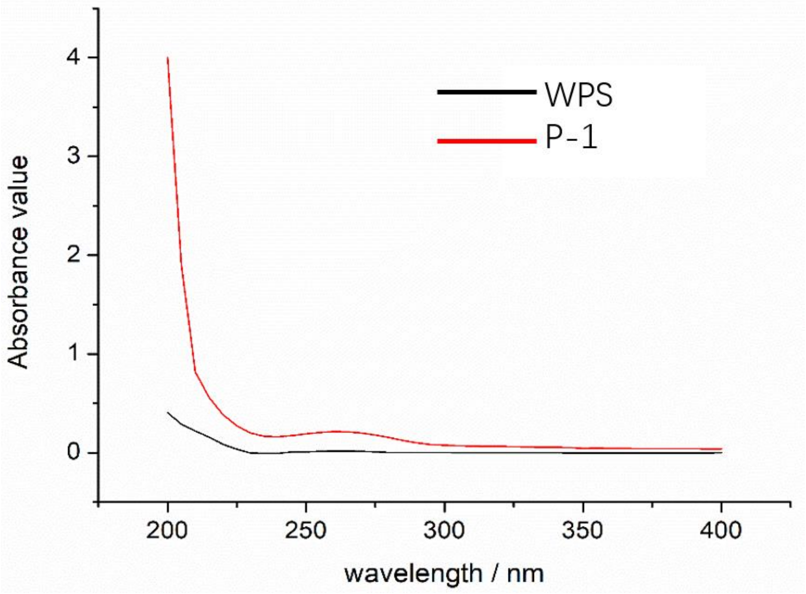

Figure S1. Ultraviolet full-wavelength scanning of PEP (WPS and P-1).

A

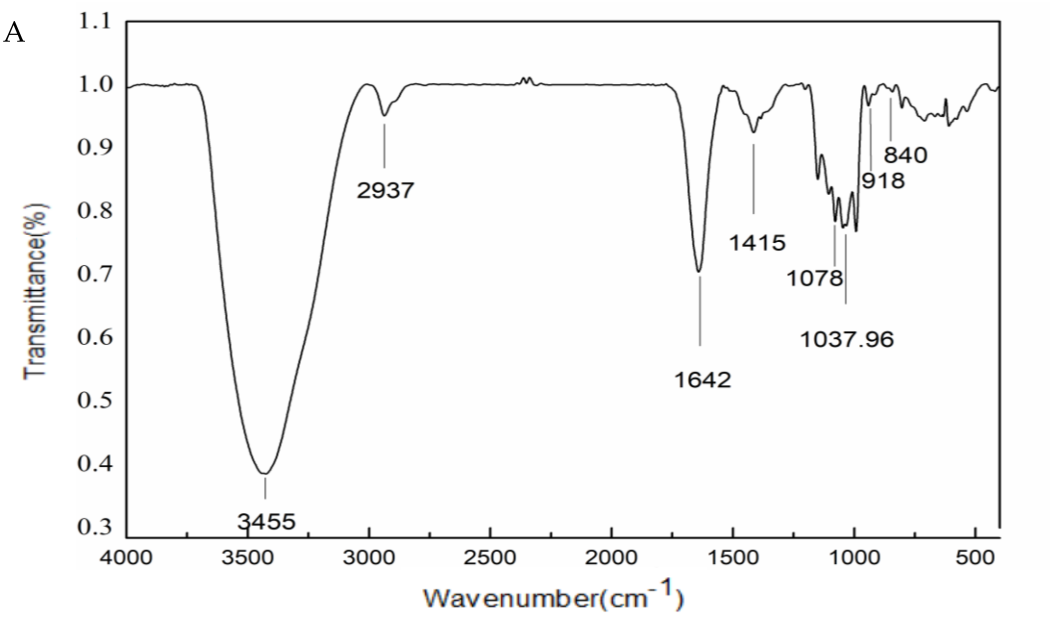

B

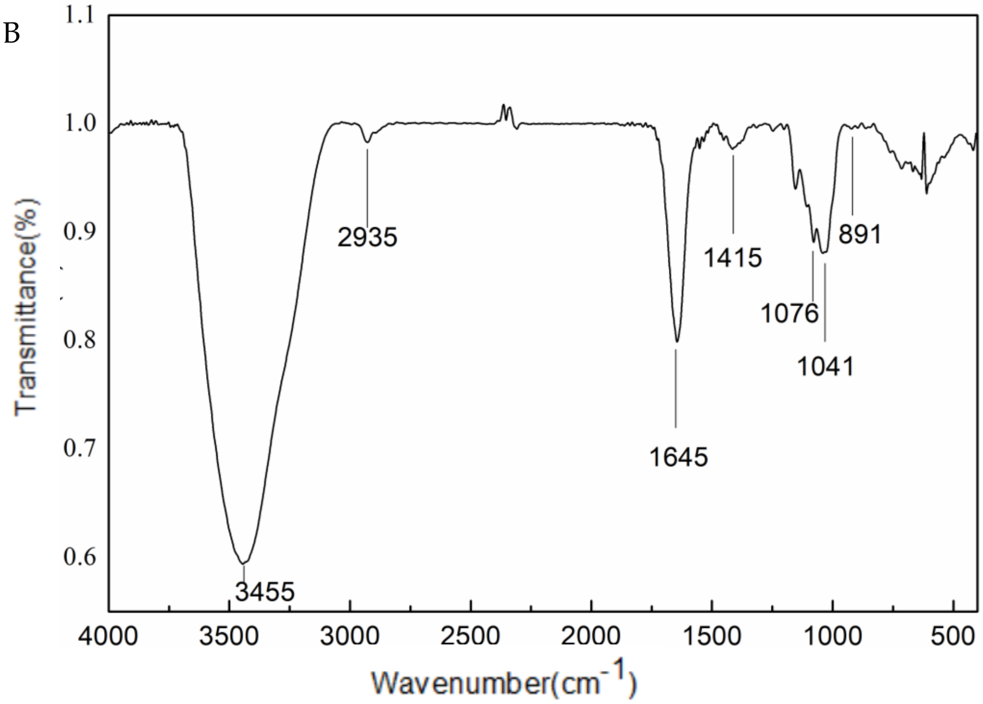

Figure S2. IR spectra of different PEPs (A) WPS. (B) P-1.

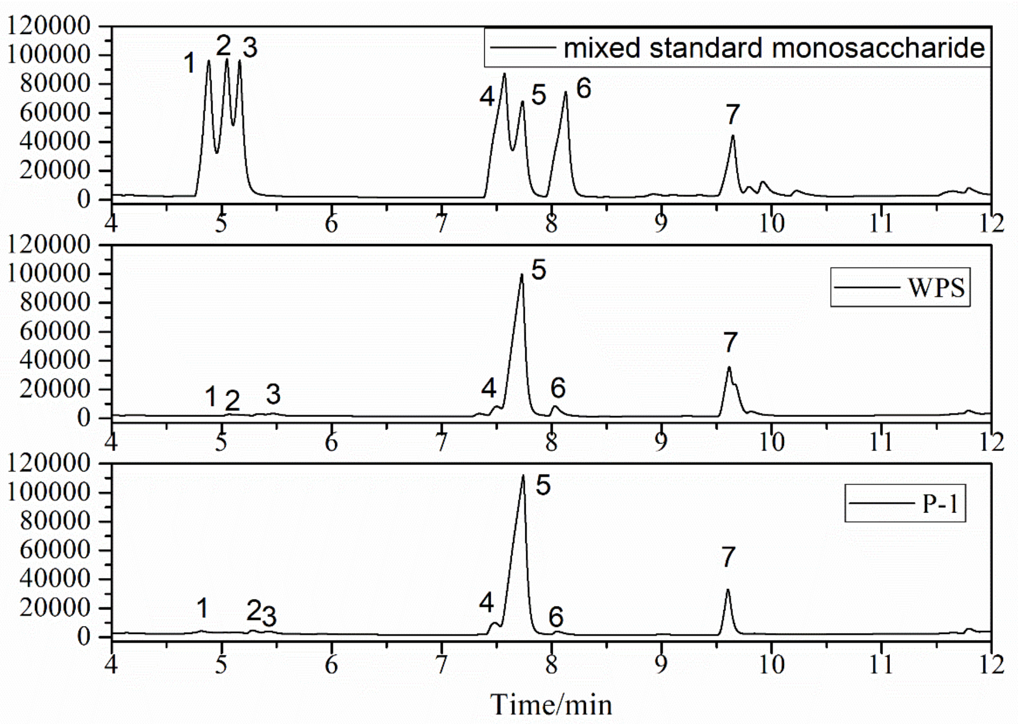

Figure S3. GC of PEPs (A)WPS. (B) P-1. (C) GC of mixed standard monosaccharide. 1. rhamnose; 2. arabinose; 3. xylose; 4. mannose; 5. glucose; 6 galactose; 7. internal standard.

A

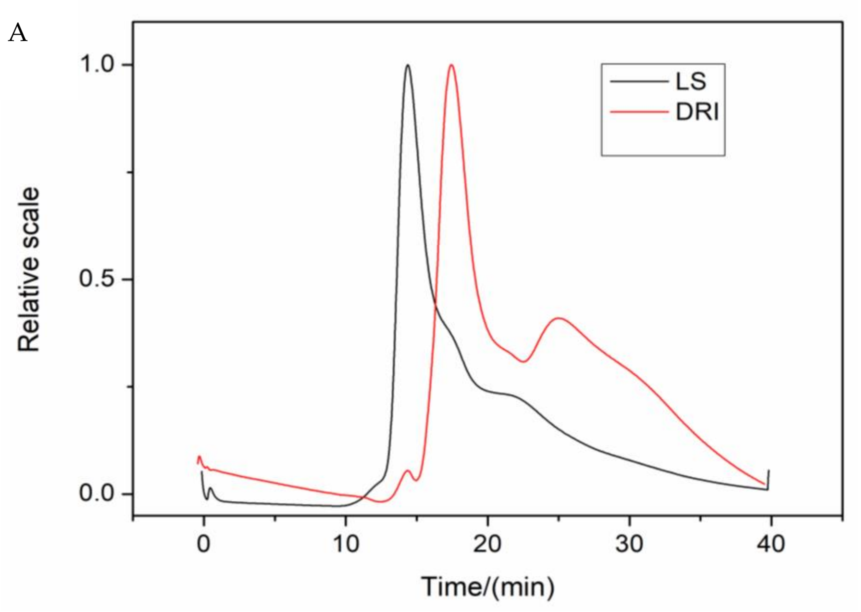

LS
DRI
Relative scale
1.0
0.5
0.0
0
10
20
30
40
Time/(min)

B

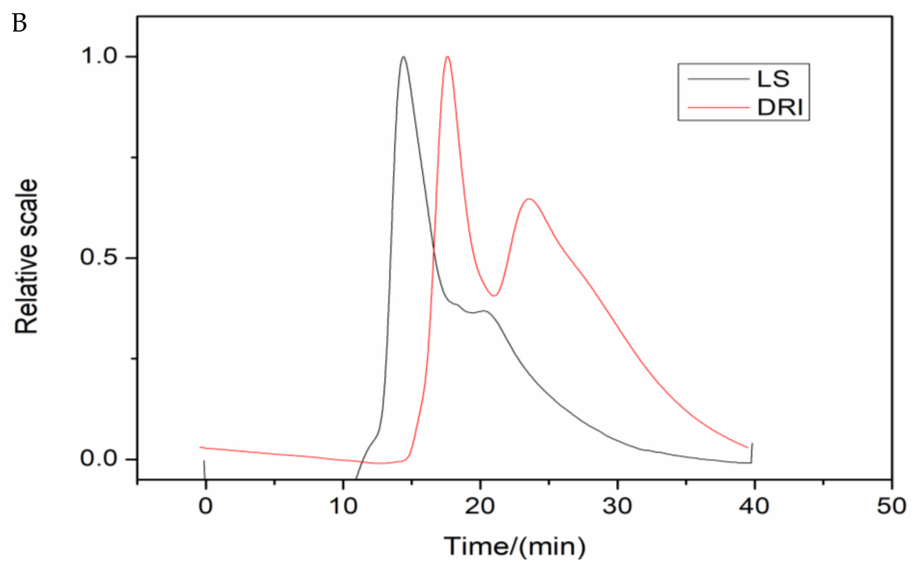

LS
DRI
Relative scale
1.0
0.5
0.0
0
10
20
30
40
50
Time/(min)

C

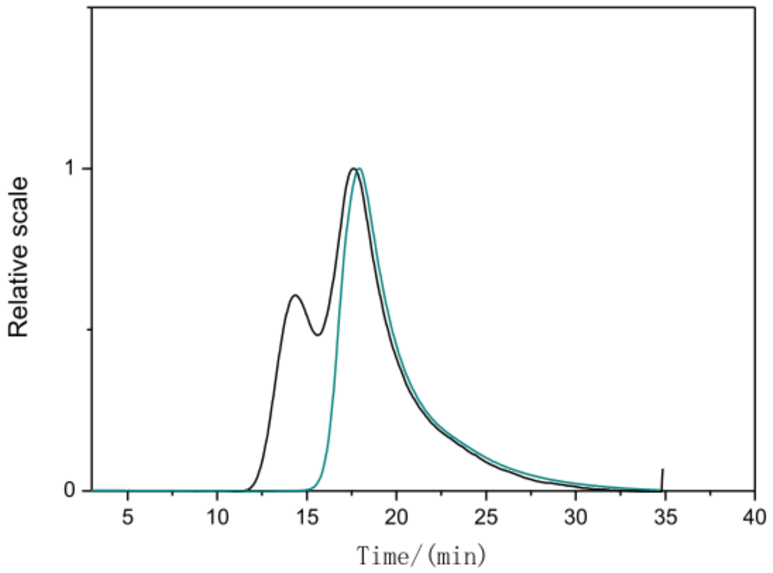

Figure S4. Gel Permeation Chromatography（GPC) spectrum of PEPs (A) GPC spectrum of WPS. (B) GPC spectrum of P-1. (C) GPC spectrum of glucan.

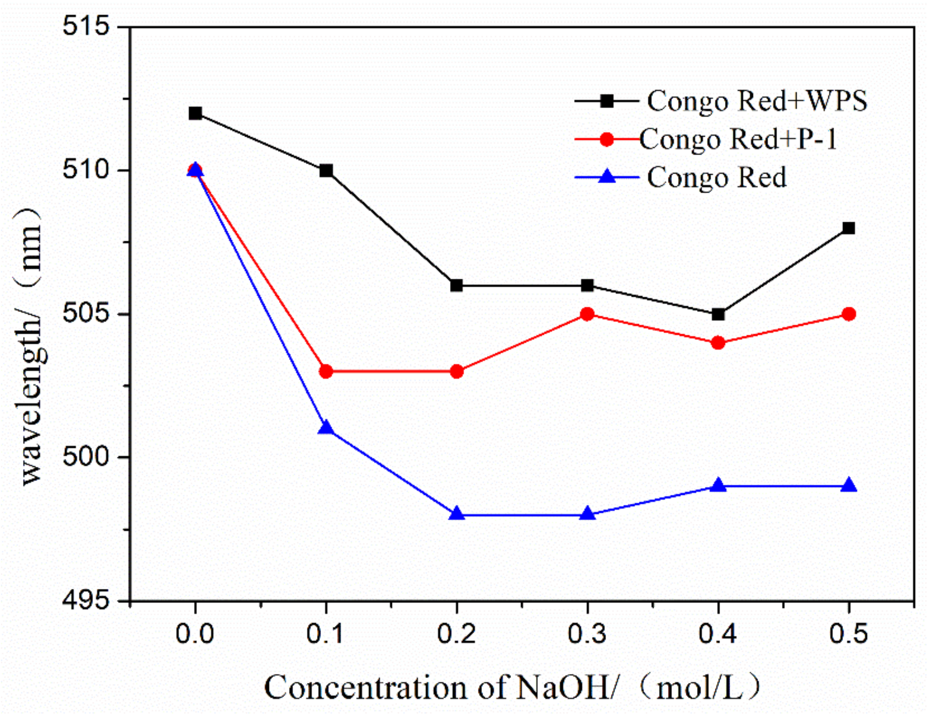

Figure S5. Congo red test of PEPs (WPS and P-1).
